# Co-inhibition of TGF-β and PD-L1 pathways in a metastatic colorectal cancer mouse model triggers interferon responses, innate cells and T cells, alongside metabolic changes and tumor resistance

**DOI:** 10.1080/2162402X.2024.2330194

**Published:** 2024-03-20

**Authors:** Reshmi Nair, Tamsin R. M. Lannagan, Rene Jackstadt, Anna Andrusaite, John Cole, Caitlin Boyne, Robert J. B. Nibbs, Owen J. Sansom, Simon Milling

**Affiliations:** a School of infection and immunity, University of Glasgow, Glasgow, UK; b Cancer Research UK Scotland Institute, Glasgow, UK; c School of Cancer Sciences, University of Glasgow, Glasgow, UK

**Keywords:** immunotherapy, colorectal cancer, T-lymphocytes, myeloid cells, Check point inhibition, metabolism, mice models, drug-mediated resistance

## Abstract

Colorectal cancer (CRC) is the third most prevalent cancer worldwide with a high mortality rate (20–30%), especially due to metastasis to adjacent organs. Clinical responses to chemotherapy, radiation, targeted and immunotherapies are limited to a subset of patients making metastatic CRC (mCRC) difficult to treat. To understand the therapeutic modulation of immune response in mCRC, we have used a genetically engineered mouse model (GEMM), “KPN”, which resembles the human ‘CMS4’-like subtype. We show here that transforming growth factor (TGF-β1), secreted by KPN organoids, increases cancer cell proliferation, and inhibits splenocyte activation *in vitro*. TGF-β1 also inhibits activation of naive but not pre-activated T cells, suggesting differential effects on specific immune cells. *In vivo*, the inhibition of TGF-β inflames the KPN tumors, causing infiltration of T cells, monocytes and monocytic intermediates, while reducing neutrophils and epithelial cells. Co-inhibition of TGF-β and PD-L1 signaling further enhances cytotoxic CD8^+^T cells and upregulates innate immune response and interferon gene signatures. However, simultaneous upregulation of cancer-related metabolic genes correlated with limited control of tumor burden and/or progression despite combination treatment. Our study illustrates the importance of using GEMMs to predict better immunotherapies for mCRC.

## Introduction

Colorectal carcinoma (CRC) is one of the most prevalent cancers worldwide and has a mortality rate of 20–30%. Metastasis of tumor to adjacent organs is the major cause of death and almost 20% of the patients diagnosed with CRC have metastasis.^1,2^ The current options for targeted therapies are very limited such as anti-Vascular Endothelial Growth Factor (VEGF), Anti-Epidermal Growth Factor Receptor (EGFR) and anti-Programmed Cell Death-1 (PD1) but only a fraction of patients respond to the therapies. Many treated individuals either do not respond to these therapies (innate resistance) or show recurrence or develop resistance to treatments (adaptive resistance).^3,4^ Patient stratification is limited to screening of PD-L1 expression for immunotherapy, and no other clinical biomarkers have been identified to differentiate responders from non-responders.^5–7^ Hence, there is a need to identify combinatorial therapies to the right subset of patients with CRC.

CRC can be differentiated into four Consensus Molecular Subtypes (CMS) based on microsatellite instability (MSI) and chromosomal instability (CIN). Driver mutations in genes like *APC, TP53, SMAD4, KRAS*, and *PIK3CA* initiate tumor development and chromosomal instability, making the tumor invasive and metastatic.^8^ In CRC, mutations in *KRAS (*40–50%), *TP53 (*43%), *and NOTCH (*16.5–25%) genes are common,^7,9,10^ and 23% tumors are CMS4 (mesenchymal) subtype.^11,12^ CMS4 tumors are characterized by *SMAD4* mutations, TGF-β upregulation, presence of endothelial cells and cancer-associated fibroblasts (CAFs), making these tumors highly metastatic with poor survival.^11,13^ Hence, it is critical to use for appropriate preclinical models resembling human CRC subtypes to test immune therapies that may improve treatment of CMS4 mCRC.

The KPN mouse model (*villin*Cre^ER^*Kras*^G12D/+^*Trp53*^fl/fl^ *Rosa26*^N1icd/+^) represents the CMS4 CRC.^14^ These genes are under the control of cre-recombinase driven by a tamoxifen-inducible *villin* promoter expressed in intestinal epithelial cells. Mutation in the *Kras* affects the RAS/MAPK pathway, resulting in uncontrolled cell proliferation^15^ Deletion of *Trp53* results in genetic instability.^16^ Constitutively active truncated Notch1 intracellular domain “N1icd” drives KPN toward an immunosuppressive TGF-β-driven tumor microenvironment (TME).^14^ The combination generates tumors in the intestine with metastases in the liver and adjoining organs.^14^ Late-stage liver metastasis is modeled using the intra-splenic transplantation. Organoids derived from KPN liver metastases are injected as single cells into the spleen, rapidly generating metastasis in the liver. Thus, these intra-splenic KPN mice provide a model to understand therapy-driven immune regulation in mCRC.

The TGF-β cytokine superfamily includes ligands TGF-β1,2 and 3.^12^ Upon activation, the inactive TGF-β1 ligand binds to the serine/threonine kinase receptors, TGF-βRI (ALK5) and TGF-βRII. This binding activates or represses target genes associated with either tumor suppression or progression.^12^ Tumorigenic effects of TGF-β include promoting epithelial to mesenchymal transition (EMT), angiogenesis, immune evasion, myofibroblast generation, and metastasis.^12,17^ TGF-β also inhibits natural killer (NK), CD8^+^T cells, M1-like macrophages, and N1 neutrophils and promotes the activity of T regulatory cells (Treg), M2-like macrophages, and N2 neutrophils near the TME, resulting in disease aggravation^18,19^ Blocking circulating TGF-β inhibits immune suppression and tumor progression. ALK5 inhibitor (ALK5i) or anti-TGF-β antibody inhibit TGF-β signaling pathway by either inhibiting phosphorylation of TGF-βRI kinase domain, or by blocking TGF-β ligand to receptor interactions^14,20,21^

Programmed cell death ligand 1(PD-L1/CD274), expressed by tumor cells and antigen presenting cells (APCs), is the ligand for Programmed Cell Death 1(PD-1/CD279), on T cells. The inhibitory interaction between PD1 with PD-L1 enables the tumor cells to escape host immune surveillance by suppressing antigen recognition, lymphocyte infiltration, and effector functions.^22^ Antibody targeting of PD-L1 inhibits PD1/PD-L1 interactions, potentially allowing T cells to proliferate, perform their effector functions, and reestablish the anti-tumor immune response.^23^

Though there have been significant advancements in precision/targeted therapy for cancer, limited information is available about the optimum treatment of CMS4 subtype of tumors with high TGF-β and activated Notch signaling. Since *KRAS, TRP53, and NOTCH1* gene mutations play a critical role in mCRC in humans, the human-CMS4 like CRC tumor characteristics in KPN makes it an interesting model to evaluate therapy driven anti-tumor immune response. We have used this model to answer (a) whether targeting TGF-β alongside with immune checkpoint blockade would help in modulating immune response and curing liver metastasis of KPN tumors and (b) what could be the underlying mechanisms of resistance if the combination therapy is not efficacious. To test this hypothesis, *in vitro* and *in vivo* experiments were conducted using KPN organoids and with the help of techniques such as flow cytometry, immunohistochemistry and TempOseq-based RNA sequencing we have investigated the mechanisms modulating the immune response and potential resistance mechanisms in mCRC after co-inhibition of TGF-β and PD-L1 signaling pathways. Our study highlights the importance of using GEM models like KPN to predict novel immune therapy combinations that may have a higher chance of translatability to the clinic.

## Results

### KPN organoids secrete TGF-β1, enhancing tumour proliferation and inhibiting splenocyte activation

**Figure 1.**
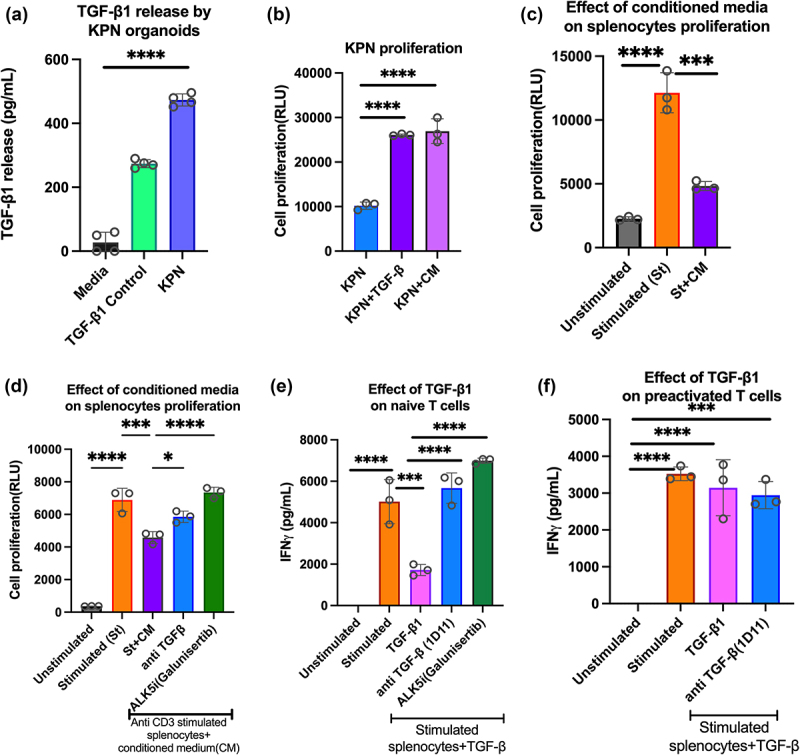
Effect of TGF-β1 on organoids and immune cells.

TGF-β signaling is characteristic of the CMS4 CRC, and Notch drives epithelial TGF-β2 expression in KPN tumors.^14^ Since TGF-β1 is the most abundant isoform,^17^ we tested its role in KPN organoids, and in inhibiting T cell activation. *In vitro*, cell culture supernatant of KPN organoids contained TGF-β1 (Figure 1(a)), and conditioned media (CM) from organoid cultures increased KPN proliferation to a similar extent as addition of recombinant TGF-β1 (Figure 1(b)). To evaluate TGF-β1-mediated immune cell suppression, anti-CD3-stimulated splenocytes were cultured with TGF-β1 or CM. Reductions in cell proliferation and IFN-γ release were observed with TGF-β1 and CM, and restored with ALK5i (Galunisertib), and anti-TGF-β antibody (1D11) (Figure 1(c,d), FigS1A-S1B). Because TGF-β1 drives both pro and antitumorigenic responses, we tested its effects on naïve and preactivated T cells. IFN-γ release and proliferation of activated splenocytes (naïve T cells) was inhibited by TGF-β1, and inhibition was blocked by anti-TGF-β antibody or ALK5i (Figure 1(e) FigS1C). In contrast, inhibition was not observed when splenocytes were pre-stimulated, rested, and restimulated with added TGF-β1 (Figure 1(f), FigS1D). Thus, CM from KPN tumors has tumor proliferative and immunosuppressive properties like recombinant TGF-β1, and TGF-β1 inhibits activation of naïve but not pre-stimulated T cells.

(a) TGF-β1 in conditioned medium (CM) from KPN organoids at 72 h. (b) KPN cells cultured with mouse TGF-β1 or CM for 72 h. (c) Anti-CD3 stimulated splenocytes (St) cultured with CM for 72 h. (d) Unstimulated or anti-CD3 stimulated splenocytes (St) cultured with CM and ALK5i or anti-TGF-β for 72 h. (e) To activate naïve cells, anti CD3 stimulated splenocytes treated with TGF-β1 and ALK5i, or anti-TGF-β for 72 h. (f) For pre-activation, anti-CD3 stimulated cells rested, restimulated with anti-CD3, with TGF-β1 and treated for 72 h. In Fig B-D, cell proliferation evaluated by cell titer glo. In Fig E-F, IFN-γ measured by ELISA. Data shown as mean±SD (*n* = 2–3). Statistical analysis: One-way ANOVA with Tukey’s multiple comparison test (Prism v9) p-value for significance *<0.05, **<0.01, ***<0.001, ****<0.0001.

### TGF-β inhibitor enhances Ly6C^+^MHCII^+^ monocytic infiltrate into KPN liver metastases

**Figure 2.**
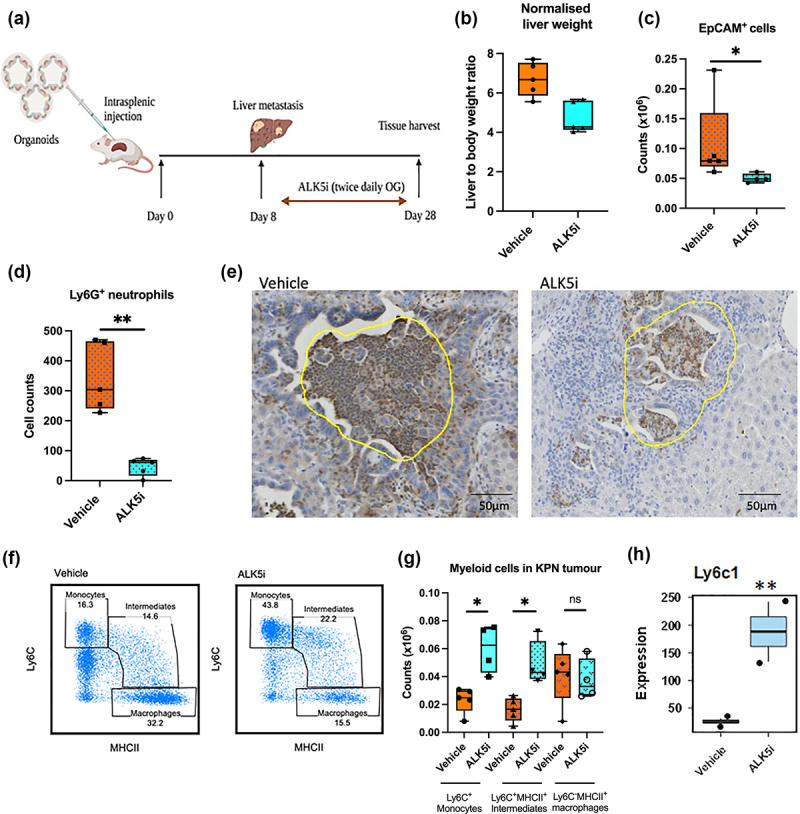
TGF-β1 inhibitor reduces neutrophils, epithelial cells and enhances inflammatory monocytes in KPN metastases *in vivo.*

To understand immune responses in KPN metastatic tumors treated with ALK5i, liver metastases were generated by intra-splenic transplantation of KPN organoids. A week after transplantation, treatment with ALK5i or vehicle was performed twice daily for 3 weeks (Figure 2(a)). Livers were then weighed and sectioned. Treatment with ALK5i reduced the liver: body weight ratio, suggesting a decrease in liver metastases (Figure 2(b)). Liver metastases were digested and stained to identify EpCAM^+^ epithelial cells and infiltrating immune cells. Flow cytometry showed that ALK5i treatment significantly reduces the number of EpCAM^+^ cells (Figure 2(c)). Immunohistochemistry (IHC) showed reduction numbers of Ly6G^+^ neutrophils (Figure 2(d,e)). A significant increase in numbers and frequency of Ly6C^+^ monocytes and Ly6C^+^MHCII^+^ monocytic intermediates occurred after ALK5i treatment (Figure 2(f,g), FigS 2A, S2B, S2C). There was no change in numbers of Ly6C^−^MHCII^+^ macrophages (Figure 2(g)). Further, RNA expression analysis by TempO-Seq also showed upregulation of *Ly6c1* after ALK5i treatment (Figure 2(h)). More CD64^+^ cells and Ly6C^+^MHCII^+^ intermediates were also observed in liver tissue of mice with low KPN metastatic tumor burden (Fig-S2D). These data indicate that KPN tumors have an inflammatory TME which is modulated after ALK5i treatment.

(a) Dosing strategy for mice with KPN metastatic tumors, treated with ALK5i or vehicle, by oral gavage (OG). (b) Normalised liver: body weight ratio between vehicle (*n* = 5) and ALK5i treated (*n* = 4). (c) Differences in EpCAM^+^ cells between vehicle and ALK5i (n same as B). (d) Counts of Ly6G^+^ neutrophils between vehicle (*n* = 5) and ALK5i (*n* = 5), from IHC. (e) Metastatic area selected for counting Ly6G^+^ cells from untreated and ALK5i treated mice(representative). (f) Flow cytometer plots and (g) counts showing Ly6C^+^ monocytes, Ly6C^+^MHCII^+^ monocytic intermediates and Ly6C^−^MHCII^+^ macrophages (n same as B). Data presented with median, minimum, and maximum values. Statistical analysis using Mann Whitney test, with p-value for significance *<0.05, **<0.01. (h) Expression of *Ly6c1* gene from metastatic area of liver FFPE sections, evaluated by TempOseq. *Ly6c1* is significant at p adj value of 0.006, represented as ** in the boxplot.

### TGF-β inhibitor enhances infiltration of lymphocytes into KPN liver metastases

**Figure 3.**
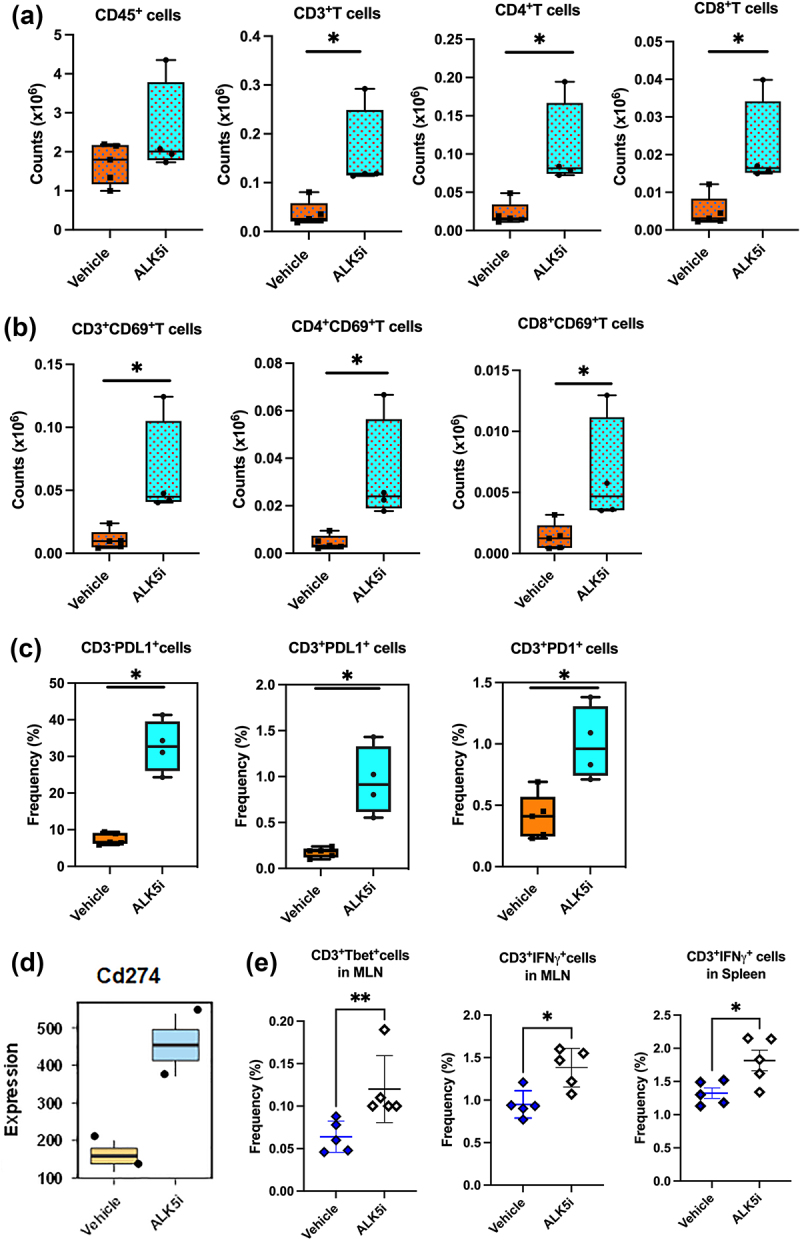
TGF-β1 inhibitor enhances lymphocyte infiltration and PD-1/PD-L1 expression into KPN metastases.

Since lymphoid cells play a critical role in anti-tumor responses, we also evaluated changes in T-lymphocytes after ALK5i treatment (Fig-S3). Increases in the numbers of tumor infiltrating CD4^+^T and CD8^+^T cells were observed after treatment (Figure 3(a)). Total CD45^+^cells did not change in frequency or numbers. The T lymphocytes were also activated, with higher CD69 expression after ALK5i treatment (Figure 3(b)). Since T cells were activated, we evaluated their expression of immune checkpoint proteins-PD1 and PD-L1. ALK5i significantly increased expression of PD-L1 on both CD3^−^ and CD3^+^ cells. Interestingly, a higher proportion of CD3^−^ cells were PD-L1^+^ than CD3^+^ cells (Figure 3(c)). TempO-Seq analysis of liver metastases also showed an increase in *Cd274 (PD-L1)* gene expression (Figure 3(d)). The tSNE plot shows an increase in the frequency of CD3^+^, CD4^+^ and CD8^+^T cells, CD69, PD1 and PD-L1 expression on T cells, and a decrease in the frequency of epithelial cells after treatment, indicating enhanced anti-tumor immune responses (Fig S4A and S4B). We also observed an increase in the frequencies of CD3^+^T-bet^+^, CD3^+^IFN-γ^+^ cells in the mesenteric lymph nodes (MLN) and spleens from KPN mice after ALK5i treatment (Figure 3(e)), indicating enhanced systemic Th1 immune responses in KPN tumor-bearing mice.

KPN liver metastases, treated with ALK5i or vehicle, were digested and stained. Live CD45^+^CD3^+^ cells were gated. (a) Counts of CD45^+^ cells, CD3^+^T, CD4^+^T and CD8^+^T lymphocytes in KPN metastases treated with vehicle control (n = 5) or ALK5i (n = 4). (b) counts of CD69^+^CD3^+^T cells, CD69^+^CD4^+^T cells, and CD69^+^CD8^+^T cells. (c) PD-L1 on CD3^−^ and CD3^+^T cells and PD1 on CD3^+^T cells between vehicle (n = 5) and ALK5i (n = 4). Statistical analysis: Mann Whitney, with p-value <0.05 considered significant (*<0.05) (Prism). (d) Boxplot of *Cd274* expression (TempOSeq), from metastatic liver FFPE sections comparing vehicle with ALK5i treatment. (e) Difference in frequencies of CD3^+^T-bet^+^ cells, CD3^+^IFN-γ^+^ in the MLN and CD3^+^IFN-γ^+^ in the spleen between vehicle (n = 5) and ALK5i (n = 5). Statistical analysis for Figures A, B, C and E was done using an unpaired Mann Whitney test. *p* value for significance *<0.05, **<0.01.

### TGF-β inhibition upregulates interferon-associated genes in KPN liver metastases

**Figure 4.**
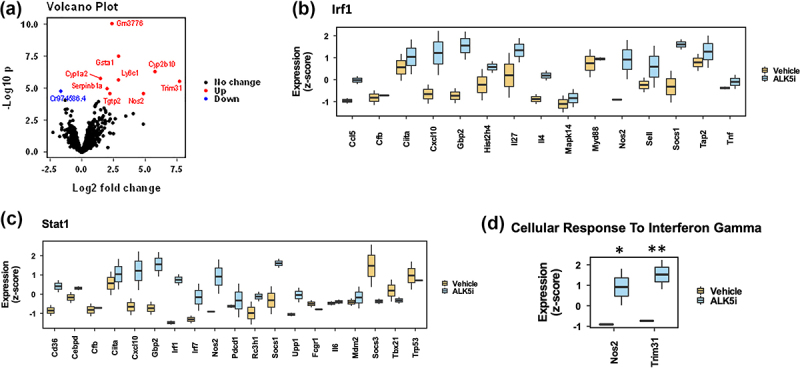
TGF-β1 inhibitor enhances expression of IRF1 associated genes.

To understand the effects of ALK5i on immune associated pathways, we analyzed formalin fixed paraffin embedded (FFPE) liver sections from metastatic tissue by whole mouse transcriptome RNA expression analysis using the TempO-Seq platform. Volcano plot shows significantly upregulated and downregulated genes between vehicle and ALK5i treatment groups (Figure 4(a)). Differential gene expression (DEG) analysis show that the genes associated with the closely related interferon regulatory factor (IRF)-1, and STAT1 pathways are enriched and most activated upstream regulators (TRUSST database) (Figures 4(b,c)). Gene ontology (GO) analysis also showed an increase in the expression of genes: *Nos2*^24^ *and Trim31*,^25^ in the Cellular_Response_to_Interferon_Gamma pathway, that are associated with modulation of tumor associated immune responses (Figure 4(d)). Thus, ALK5i enhances IRF and STAT1 gene expression, and enhances anti-tumor immune responses.

TempO-Seq results from metastases from liver FFPE sections after vehicle or ALK5i treatment. (a) Volcano plot representing differentially expressed genes between the two treatment groups (significantly upregulated genes in red, downregulated genes in blue and no change in black). (b-c) Changes in gene expression of IRF1 and STAT1 pathway associated genes (most activated upstream regulators, TRUSST database) between vehicle (*n* = 2) and ALK5i (*n* = 2). (d) Genes associated with cellular response to interferon gamma are significantly upregulated after ALK5i treatment (*Nos2* p-adj value 0.03 represented as * and *Trim31* p-adj value 0.006 represented as **)

### Co-inhibition of TGF-β and PD-L1 boosts infiltration of CD8^+^ T cells in KPN liver metastases

**Figure 5.**
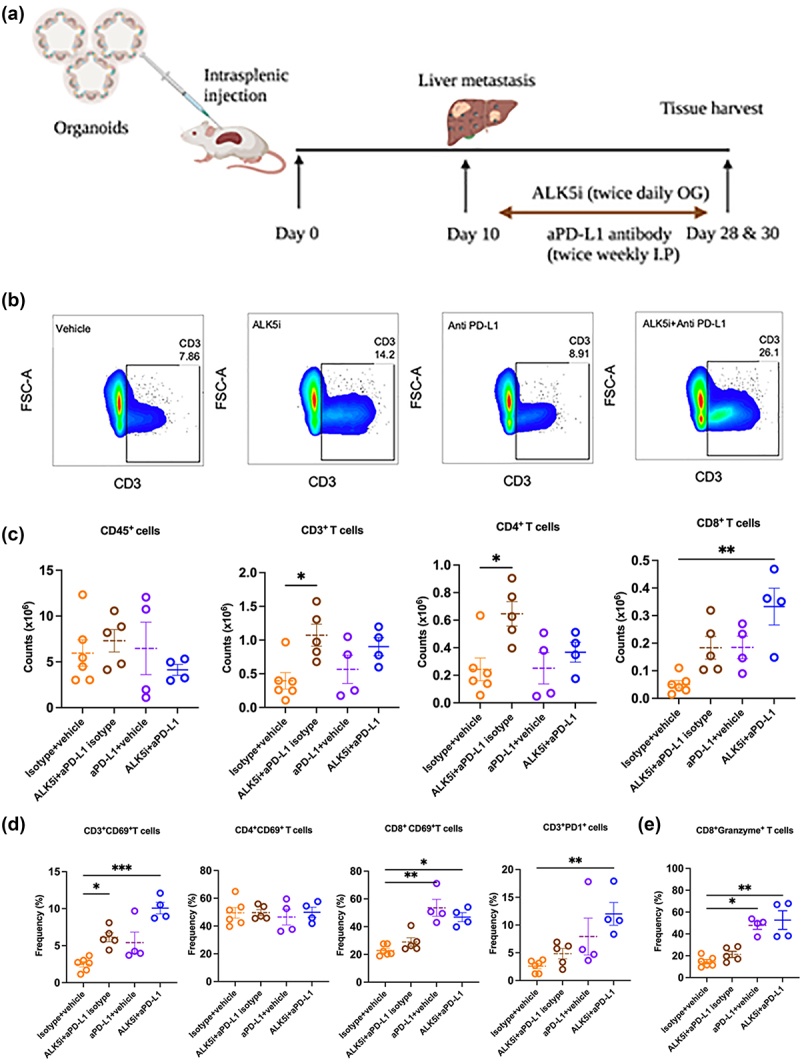
Inhibition of TGF-β and PD-L1 boosts infiltration of CD8^+^T cells.

The PD1/PD-L1 axis is often associated with T cell exhaustion, leading to immune evasion and tumor progression.^23^ Since ALK5i treatment of KPN metastases led to high PD1/PD-L1 expression but did not completely inhibit tumor growth, we hypothesized that dual inhibition of TGF-β and PD1/PD-L1 would further reduce KPN metastasis. KPN metastases were therefore treated with combination of ALK5i and anti-PD-L1 antibody, or with the individual treatments, or appropriate vehicle/isotype controls (Figure 5(a)). Samples were analyzed at endpoint by flow cytometry. Frequencies of CD3^+^T cells are shown in Figure 5(b). No changes were observed in the counts of CD45^+^ leukocytes (Figure 5(c)). ALK5i-treated animals showed a significant increase in counts of CD3^+^T cells and CD4^+^T cells over vehicle-treated mice. The combination treatment also caused a significant increase in the frequency of CD3^+^T cells over the vehicle. No changes were observed in CD3^+^ or CD4^+^T cell frequency or counts after anti-PD-L1 treatment (Figure 5(c)). Unlike CD4, CD8^+^T cells did not significantly increase in number after ALK5i treatment. However, Anti-PD-L1 single and combination treatments showed significant increases in CD8^+^T cells in the KPN tumor (Figure 5(c)).

(a) Schematic representation of KPN metastases treated with vehicle, single and combination treatments. (b) Dot plots showing frequencies (%) of CD3^+^T cells. (c) Counts of CD45^+^, CD3^+^, CD4^+^ and CD8^+^T cells between vehicle (*n* = 6), ALK5i + isotype (*n* = 5), anti-PD-L1+ vehicle (*n* = 4) and ALK5i + anti-PD-L1 (*n* = 4). (d) CD69^+^CD3^+^, CD69^+^CD4^+^, CD69^+^CD8^+^T cells, and PD1^+^CD3^+^T cells (n as for C) in the four groups. (e) Granzyme B^+^CD8^+^T cells between treatments (n as for C). Data plotted as mean ± SEM. Statistical analysis: Kruskal Wallis with Dunn’s multiple comparison test. All treatment groups are compared to vehicle, and *p* < 0.05 considered significant (*<0.05, **<0.01, ***<0.001).

We also evaluated activation of these infiltrating T cells, by assessing their CD69 expression. ALK5i alone and in combination with anti-PD-L1 caused a significant increase in the frequency of CD69^+^CD3^+^T cells and CD69^+^CD8^+^T cells, indicating CD8^+^T cell activation (Figure 5(d)) The combination treatment also elevated PD1 expression on CD3^+^T cells (Figure 5(d)). Intracellular cytokine staining showed higher frequency of granzymeB^+^CD8^+^T cells after anti-PD-L1 single, and combination treatment with ALK5i (Figure 5(e)). This increase in the frequency of CD8^+^PD1^+^, CD8^+^CD69^+^ and granzymeB^+^CD8^+^T cells was also observed in livers of mice with low KPN metastatic tumor burden (Fig-S5A-5D). Overall, co-inhibition of TGF-β and PD1/PD-L1 signaling increases infiltration of granzymeB-expressing cytolytic CD8^+^T cells into KPN metastatic tumors.

### Co-inhibition of TGF-β and PD-L1 upregulates innate immune responses in the KPN liver metastases

**Figure 6.**
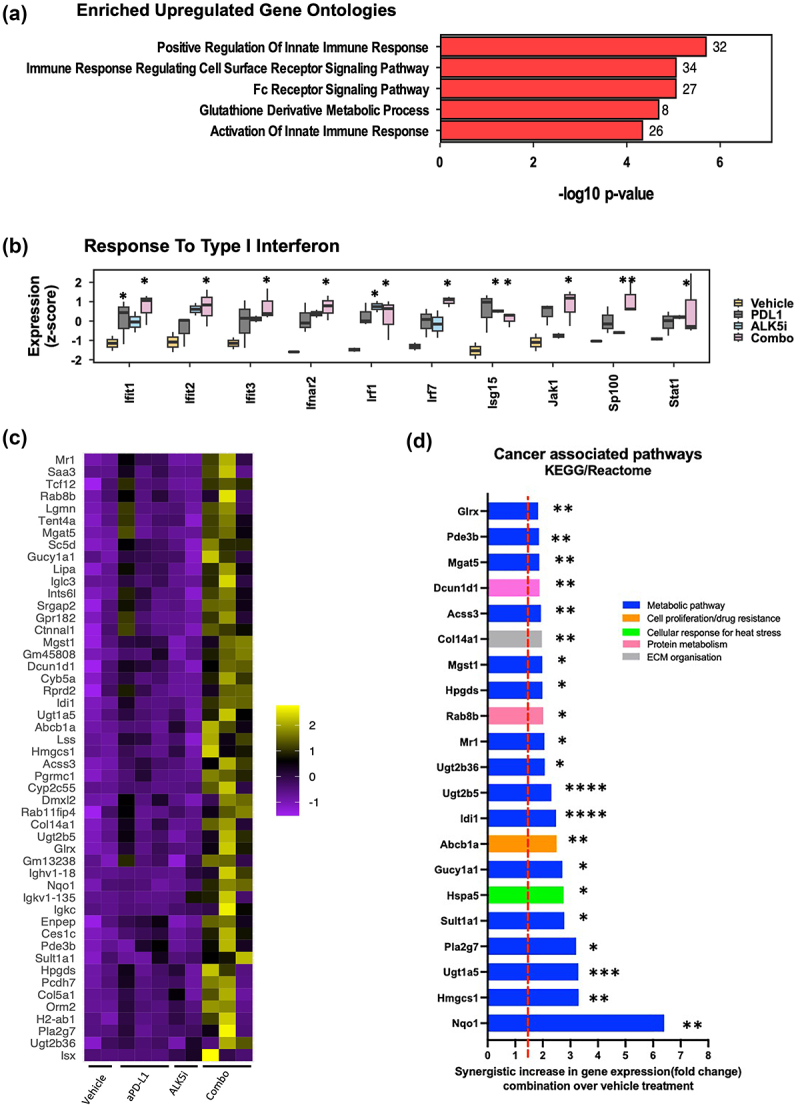
Co-inhibition of TGF-β and PD-L1 upregulates IRF-1 and cancer associated metabolic pathway genes.

To investigate mechanisms modulating the immune response in KPN tumors after combination treatment, we performed RNA sequencing of metastases from FFPE liver sections by TempO-Seq. We observed differential expression of genes (DEG) between vehicle, single and combination treatment groups (Fig-S6A-C, supplementary table S1). Since DEG were highest between vehicle and combination groups, pathway analysis was used to compare between them. This showed an increase in genes regulating and activating innate_immune_response, cell_surface_receptor_signalling, Fc_receptor_signalling, and metabolic_processes and cellular response to interferon gamma (Figure 6a, S6D-F). Thus, co-inhibition of TGF-β and PD-L1 pathways boosts the interferon gene expression in KPN metastases (Figure 6(b)).

Mice with KPN metastases treated with vehicle + isotype (n = 2), ALK5i + isotype (n = 2), anti-PD-L1 + vehicle (n = 3), or ALK5i + anti-PD-L1 (n = 3). TempO-Seq sequencing performed with metastases from liver FFPE sections. (a) 5 most enriched GO pathways comparing combination treatment to vehicle and (b) response to Type1 interferon associated genes. * Represents adjusted *p* value *< 0.05 and **<0.01 (treatment groups compared to vehicle). (c) Heatmap and boxplot showing top 50 genes synergistically upregulated after combination treatment. Clustering shown is hierarchical. (d) Bar graph showing ratio observed/expected and associated pathways for synergistic genes, evaluated using KEGG and Reactome pathway analyses. P adj value *<0.05, **<0.01, ***<0.001 and ****<0.0001 (combination treatment vs vehicle).

### Upregulation of cancer associated metabolic pathway genes associates with tumour resistance after dual treatment

Though we observed increases in both infiltration of CD8^+^T lymphocytes and IFN gene expression, there was no observed reduction of met counts, liver to body weight ratio, or in the proportion of epithelial cells after combination treatment (data not shown). Thus, we investigated potential mechanisms underlying this tumor resistance. We evaluated genes that were upregulated after combination treatment (Figure 6(b,c)) and identified the KEGG and Reactome pathways relating to the top 50 upregulated genes. The majority of the genes synergistically upregulated by inhibition of both PD-L1 and TGF-β were associated with metabolic pathways (*Nqo1, Hmgsc1, Sult1a1, Pla2g7, Ugt1A5, Ugt2B5, Abcb1a*), that enhance cancer cell proliferation, invasion, and metastasis (Fig-6D). This upregulation of cancer-related metabolic genes correlated with limited control of tumor burden, despite the combination treatment. Our data therefore indicate that the combination of ALK5i with anti-PD-L1 potentially induces tumor resistance, and correlates with upregulation of cancer-associated metabolic pathways in the KPN tumor.

## Discussion

mCRC has a 5-year overall survival of 14% and almost 50% of patients undergoing surgery develop metastasis within these 5 years.^5,26^ Hence, there is significant unmet clinical need. The KPN model provides an investigational tool to understand mechanisms modulating therapy-induced immune responses, to help develop better immunotherapy combinations to treat CMS4 type mCRC.

Jackstadt et al. has reported comparable TGF-β1 expression in both primary tumors and organoids derived from KPN mice.^14^ TGF-β1 secreted by KPN organoids has a direct effect on KPN tumors, enhancing proliferation, and inhibiting IFN-γ release by activated T cells. Immunosuppressive effect of TGF-β was primarily on naive and not pre-activated T cells. Similarly, TGF-β inhibits proliferation of naïve T cells but not antigen experienced cells in diabetes.^27^ However, a contrary report showed TGF-β1 attenuated effector function of tumor antigen-specific human memory CD8^+^T cells.^28^ The effects of TGF-β on naïve and preactivated T cells could be different due to downregulation of TGF-βRII expression on activated T cells.^29^ Thus, the timing of TGF-β inhibition and the mode of T cell stimulation play critical roles in determining its pleiotropic effects.

Since KPN tumors resemble the CMS4-CRC with high TGF-β, we evaluated the effect of ALK5i on myeloid cells in these tumors. High numbers of Ly6C^+^ monocytes and Ly6C^+^MHCII^+^ monocytic intermediates reveal an inflammatory TME. This increase in monocytic intermediates is like that observed in the inflamed intestine.^30^ TGF-β is also indispensable for monocyte to macrophage differentiation in the intestine and could also play this role in tumors.^31^ Neutrophils contribute to inflammation and metastasis, and a decrease in Ly6G^+^ neutrophil after ALK5i treatment modulates inflammatory TME.

ALK5i treatment enhanced T cell infiltration into tumors, increasing numbers of activated CD69^+^CD4^+^ and CD8^+^T cells. In addition, fewer EpCAM^+^ epithelial cells indicated a reduction in tumor cellularity. TempO-seq analysis confirmed upregulation of IRF-associated genes in the tumor and enhanced systemic Th1 immune responses. Therefore, inhibiting TGF-β in KPN tumors enhances the anti-tumor immune response.

Despite the ALK5i-mediated enhancement in infiltrating T cells and inflammatory myeloid cells, treatment was not highly effective. The T-lymphocytes showed high expression of exhaustion markers PD1 and PD-L1. Exhaustion of T cells could enable tumor resistance, so we treated tumors with a combination of ALK5i and anti-PD-L1. This significantly increased the activated CD8^+^T cells, and enhanced expression of granzymeB^+^CD8^+^T cells. Our data indicate that combination treatment of KPN tumors with ALK5i and anti PD-L1 antibody skews toward Type 1 interferon and interferon gamma mediated immune response by innate cells and CD8^+^CTLs. These cells are often associated with improved survival of colorectal and other cancers^32.^

TempO-Seq analysis showed upregulation of innate immune response and IRF pathway genes *(IRF1*, *IRF7, IFNAR2, JAK1, STAT1)*, yet no reduction was observed in the metastatic tumors. Thus, the combination of ALK5i and anti-PD-L1 was insufficient to eliminate metastatic KPN tumors. An increase in the gene expression of *Nos2* was observed in the combination treatment group when compared to vehicle. *Nos2* has a dichotomous role in modulating tumor immune response as it is induced by interferon gamma and is often associated with tumorigenesis and poor prognosis in cancer.^33,34^ In addition to *Nos2*, an increase in the expression of cancer-associated metabolic genes (*Nqo1, Hmgsc1, Pla2g7, Sult1a1, Ugt1A5, Ugt2B5, Abcb1a)* indicates reprogramming of tumor metabolic pathways, potentially leading to the observed tumor resistance.^35–42^ *Abcb1 encodes* the ABC transporter protein, which confers multidrug resistance in cancer, fosters cancer stem cell-like properties, and facilitates epithelial–mesenchymal transition.^43^ Interestingly, inhibiting ABCB1 overcomes acquired resistance of non-small cell lung cancer cells to MET inhibitors.^41^

The metabolic genes, *Nqo1, Hmgcs1, Pla2g7, and Sult1a1*, encode enzymes such as cytosolic reductase, mevalonate, phospholipase, and sulfotransferase. Like *Abcb1*, upregulation of these genes is closely linked with the advancement of cancer and its metastatic spread.^35,37,39,42^ Some of these metabolic genes modulate anti-tumor immune responses. For instance, *Sult1a1* contributes to immune exclusion, which manifests as a negative correlation with crucial immune cell populations including CD8+ cells, CD4+ cells, macrophages, and neutrophils.^42^ Furthermore, therapeutic targeting of NQO1 and HMGCS1 can augment T-cell responses by fostering pyroptosis, an inflammatory form of cell death, and ultimately enhancing anti-tumor immunity.^44,45^ Recent findings suggest that PLA2G7^high^ macrophages create an immunosuppressive microenvironment in the tumor and hinder CD8 T-cell activation, and its inhibition enhances efficacy of anti-PD1 treatment.^46^ These findings highlight the therapeutic potential of modulating metabolic pathways to boost the immune response against cancer.

Another colon cancer metastasis mouse model, AKPT, showed tumor regression after inhibition of TGF-β and PD-1/PD-L1.^19^ Enhanced Notch 1 activation and neutrophil infiltration differentiates the KPN model used here from other CRC models, including AKPT, and these factors may influence the therapeutic response. In a phase II trial for CMS4 mCRC, patients received bintrafusp alfa, a dual PD-L1 antibody/TGFβ trap alongside radiation. Though changes in IFN-γ signature was observed, efficacy was low.^47^ This correlates with our observation of combination treatment in the KPN tumors. Therefore, aggressive mCRC will require additional treatment along with TGF-β and PD-L1 inhibitors. GEMMs like KPN, resembling human CRC,^48^ will continue to be helpful in evaluating pre-clinical efficacy and immune modulatory mechanisms in hard-to-treat metastatic colorectal cancer.

## Materials and methods

### KPN organoid culture

KPN liver metastasis organoids were generated as previously described.^14^ Organoids were cultured in Base Medium including Supplements (BMS), with 50 ng/mL Human EGF and 100 ng/mL murine noggin (Peprotech, USA). Organoids were mixed with Growth Factor Reduced Matrigel (Corning, USA) before seeding. Organoids were frozen in Recovery cell culture freezing medium (ThermoFisher, USA) and thawed as required.

For *in vitro* assays, organoids (BVKPN RKAC13.1e) were trypsinized with TrypLE and washed with 1 mL FACS buffer (PBS +2%FBS +1 mM EDTA). For flow cytometry, cells were stained with fixable viability dye (FVD) (ThermoFisher, USA) and anti-mouse EpCAM (clone G8.8, Biolegend, USA). Supernatant from KPN organoid culture at 72 h was used for TGF-β1 estimation using ELISA (R&D systems, USA). For proliferation assay, KPN cells were washed and seeded in BMS medium. Recombinant mouse TGF-β1 (0.5 ng/mL, R&D systems, USA) or conditioned medium (CM), from organoids cultured for 72 h, was added and proliferation measured after 72 h using cell titer glo (Promega, USA).

### Splenocyte proliferation assay

Splenocytes were harvested from C57BL/6 mice and frozen in RPMI with 90%FBS (Gibco, USA) and 10% DMSO (Sigma, USA). On the day of the assay, frozen cells were revived in RPMI media containing 10% FBS and seeded in a 96 well plate as 1–2×10^5^ million cells/well. The cells were stimulated (St) using plate-coated anti-CD3 (0.5 μg/mL) in the presence of either TGF-β1 (0.5 ng/mL), or 50 μL of CM. Some wells were supplemented with anti-TGF-β1 antibody (10 μg/mL, Bioxcell, USA) or ALK5i (Galunisertib,10 μM, Tocris, U.K). After 72 h, proliferation was measured by cell titer glo (Promega, USA) and cytokine release by IFN-γ ELISA (R&D systems, USA).

### T cell re-stimulation assay

To assess the effects on previously activated T cells, frozen splenocytes seeded in anti-CD3 coated (0.5 μg/mL) plates. After 24 h, cells were washed and rested overnight. Cells were re-seeded for anti-CD3 re-stimulation and treated as described above.

### Intra-splenic model of metastatic cancer and treatments

Mouse experimental work was carried out in accordance with UK Home Office regulations (Project Licenses 70/8646 and 70/9112), adhered to ARRIVE guidelines, and were subject to ethical review at the University of Glasgow. Injection of liver metastatic KPN organoids were performed as previously described^14^ on C57BL/6 mice, 6–7 weeks old (Charles River, UK) under specific pathogen-free conditions. Mice received a single intra-splenic injection of 5 × 10^5^ BVKPN RKAC3.2f cells in 50 µL of phosphate buffer saline (PBS).

### ALK5i treatment of KPN metastatic tumour bearing mice

One week after intra-splenic injection mice received either vehicle (*n* = 5) or ALK5i treatment (*n* = 5). 50 mg/kg of ALK5i (AstraZeneca AZ12601011) was dosed twice daily by oral gavage in 100 µL of 0.5% Hydroxypropyl Methylcellulose (HPMC) and 0.1% Tween-80 (vehicle). After 3 weeks the mice were euthanized and tissues were collected for analysis.

### ALK5i and anti-PD-L1 treatment

One week after intrasplenic injection, mice received (a) rat IgG2b isotype antibody (Bioxcell BE0090) and vehicle (0.5% HPMC/0.1% Tween-80) (*n* = 6), (b) ALK5i and isotype antibody (*n* = 6), (c) anti-PD-L1 antibody (Bioxcell BE0101) and vehicle (*n* = 6), or (d) combination of ALK5i and anti-PD-L1 antibody (*n* = 6). Anti-PD-L1 and isotype antibody were injected at 10 mg/kg intraperitoneal (IP) twice per week. After 3 weeks, mice were euthanized and tissues were collected for analysis.

### Tumour digestion flow cytometry

Macroscopic tumor metastases were dissected from liver tissue, minced (~0.5 mm), and then digested for 30 minutes using 1.5 mg/mL Collagenase V, 0.1 mg/mL of Hyaluronidase (Sigma, USA) and 100 µg/mL DNase I (Roche, Switzerland) in RPMI complete medium. Samples were filtered and the enzyme was neutralized. 1 × 10^6^ cells from each sample were used for Fc blocking (Biolegend, USA) at 4°C followed by staining for a lymphoid panel: viability dye (Thermo, USA), anti-mouse CD45 (30-F11), CD3 (17A2), CD4 (RM4–5), CD8 (53–6.7), CD69 (H1.2F3), PD-1 (29F.1A12), PD-L1 (10F.9G2) and EpCAM (G8.8). For the myeloid panel: viability dye, anti-mouse CD45 (30-F11), B220 (RA3-6B2), CD64 (X54–5/7.1), Ly6C (HK1.4) and MHC-II (M5/114.15.2). All antibodies were from Biolegend, USA. Cells were washed and resuspended in FACS buffer and acquired using BD Fortessa/LSRII/Aria.

### Spleen and lymph node digestion

Spleen and mLN were harvested, chopped into fine pieces, and digested using collagenase D (1 mg/mL, Sigma, USA) in complete RPMI, for 25 min at 37°C. Cells were filtered and washed with FACS buffer. RBC lysis was carried out for spleen cells using ACK lysis buffer (Sigma, USA).

### Transcription factor staining

2×10^6^ cells were Fc blocked (Biolegend) at 4°C for 20 min, washed, and stained with viability dye (Thermo, USA), anti-mouse CD45 and CD3 (Biolegend, USA). The cells were washed and fixed with fixation/permeabilization reagent (Thermo, USA) for 30 min at RT, washed with permeabilization buffer (Thermo), and stained with anti-mouse T-Bet (4B10, Biolegend, USA).

### Intracellular staining

2×10^6^ cells were resuspended in complete RPMI with cell stimulation cocktail (Thermo, USA). After washing and Fc blocking, they were stained with viability dye (Thermo, USA), anti-mouse CD45 (30-F11), CD3 (17A2), CD4 (RM4–5), CD8 (53–6.7), CD69 (H1.2F3), (Biolegend, USA). Cells were washed, fixed and permeabilized (Thermo, USA), and were stained with anti-mouse IFN-γ (XMG1.2) and granzyme B(QA16A02) (Biolegend, USA). Cells were washed and resuspended in permeabilization buffer before acquisition using BD Fortessa or LSRII flow cytometers.

### Analysis

Flowjo v10.8.1 was used for the analysis of flow cytometry data.

### Immunohistochemistry (IHC)

4 µm FFPE sections of liver with metastases were H&E stained, using standard methods. For Ly6G detection, FFPE sections were deparaffinized and hydrated using xylene, graded alcohol and rinsed with distilled water. Endogenous peroxide was quenched using Bloxall (Vector labs). 0.01 M citrate buffer pH 6.0 was used for antigen retrieval. Anti-mouse Ly6G (Bioxcell BE0075–1), ImmPRESS reagent (Vector Lab), rewashed, and Impact DAB chromogen solution were used to visualize Ly6G^+^ cells. Sections were counter stained with Haematoxylin. Metastatic areas (~4×10^5^µm^2^) were analyzed to count Ly6G^+^ cells using Qupath v0.3.2.

### RNA sequencing by TempO-seq

Tumour areas from FFPE liver sections were excised for targeted sequencing-based RNA expression analysis using Tempo-Seq. Metastatic regions from liver sections were pooled (2–3 mice/group) for sequencing. Differential gene expression was calculated using DESEQ2 (1.38.3). Data were explored using Searchlight^49^(V2.0,0) specifying DE workflow for Vehicle vs PDL1, Vehicle vs ALK5i, and Vehicle vs Combination, and one MDE workflow combining the 3DE. Significance threshold is Wald test with BH correction, adjusted *p* < 0.05. Over-representation analysis used GO mouse biological process database and TRUSST mouse database for upstream regulator analysis.

Synergistic response of combination treatment over vehicle was calculated using formula: {expected = mean (ALK5i-Vehicle) + (aPDL1-Vehicle), observed = mean (combination), synergy = ratio of observed/expected}, and confirmed using Bliss analysis (E_AB_=E_A_+E_B_-E_A_*E_B_), where E_A=_ALK5i and E_B=_anti PD-L1 and E_AB_ is the additive effect. For a gene to be synergistic, counts in combination treatment group > E_AB_.

## Statistical analysis

*In vitro* data were analyzed as mean±SD and statistical analyses was performed using One-way ANOVA and Tukey’s multiple comparison test. Statistical analysis between two *in vivo* groups was done using an unpaired Mann Whitney test. Data between four treatment groups were analyzed using Kruskal Wallis with Dunn’s multiple comparison test. Analysis was performed with GraphPad Prism software and p-value *<0.05 was considered significant. Sample sizes are included in figure legends.

## Supplementary Material

Supplemental Material

## Data Availability

The data generated in this study are available upon request from the author.
