# Supplementary figures and images for: Co-inhibition of TGF-β and PD-L1 pathways in a metastatic colorectal cancer mouse model triggers interferon responses, innate cells and T cells, alongside metabolic changes and tumor resistance
Source: Oncoimmunology. 2024 Mar 20;13(1):2330194. doi: 10.1080/2162402X.2024.2330194 (PMC10956632; doi:10.1080/2162402X.2024.2330194)

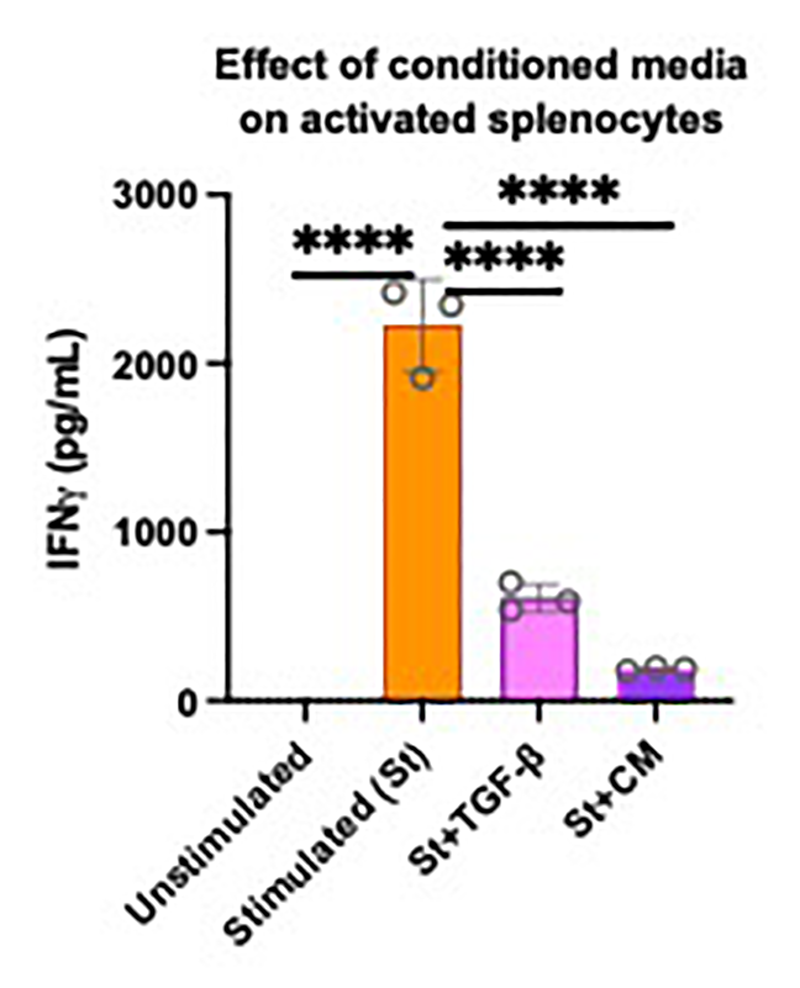

Supplement: Supplemental Material [file KONI_A_2330194_SM7783.zip › SupFig1A..tiff]

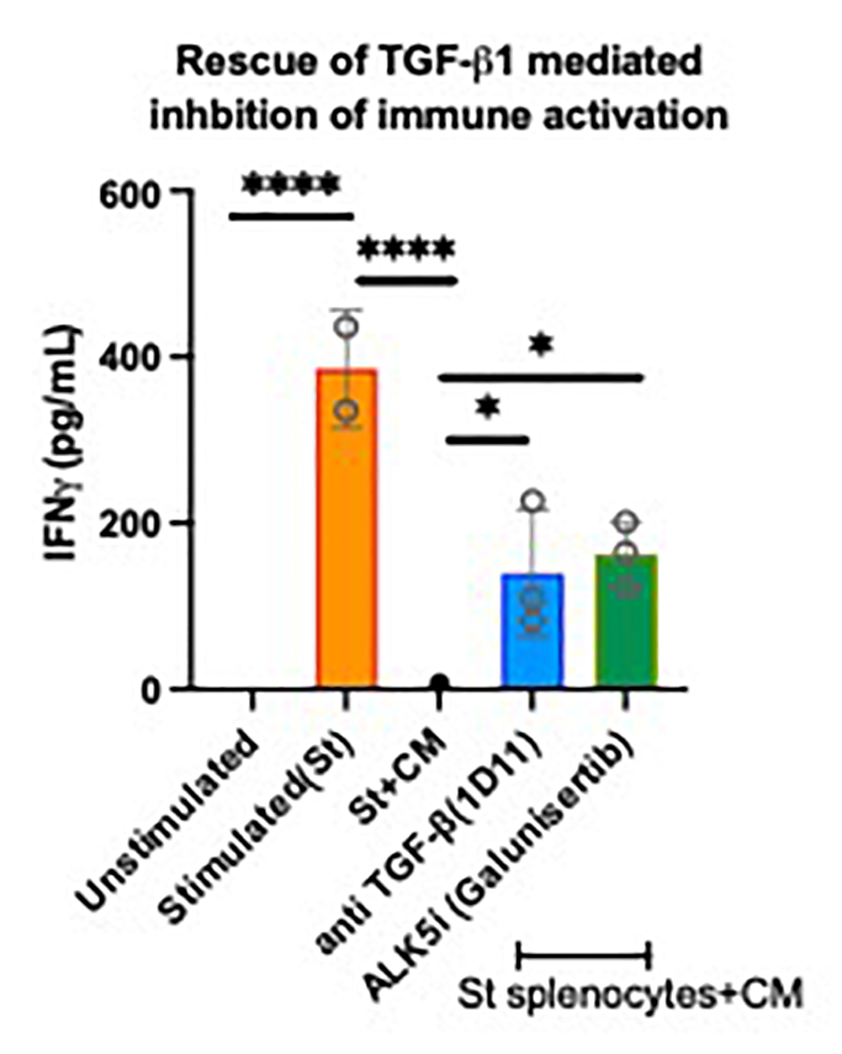

Supplement: Supplemental Material [file KONI_A_2330194_SM7783.zip › SupFig1B..tiff]

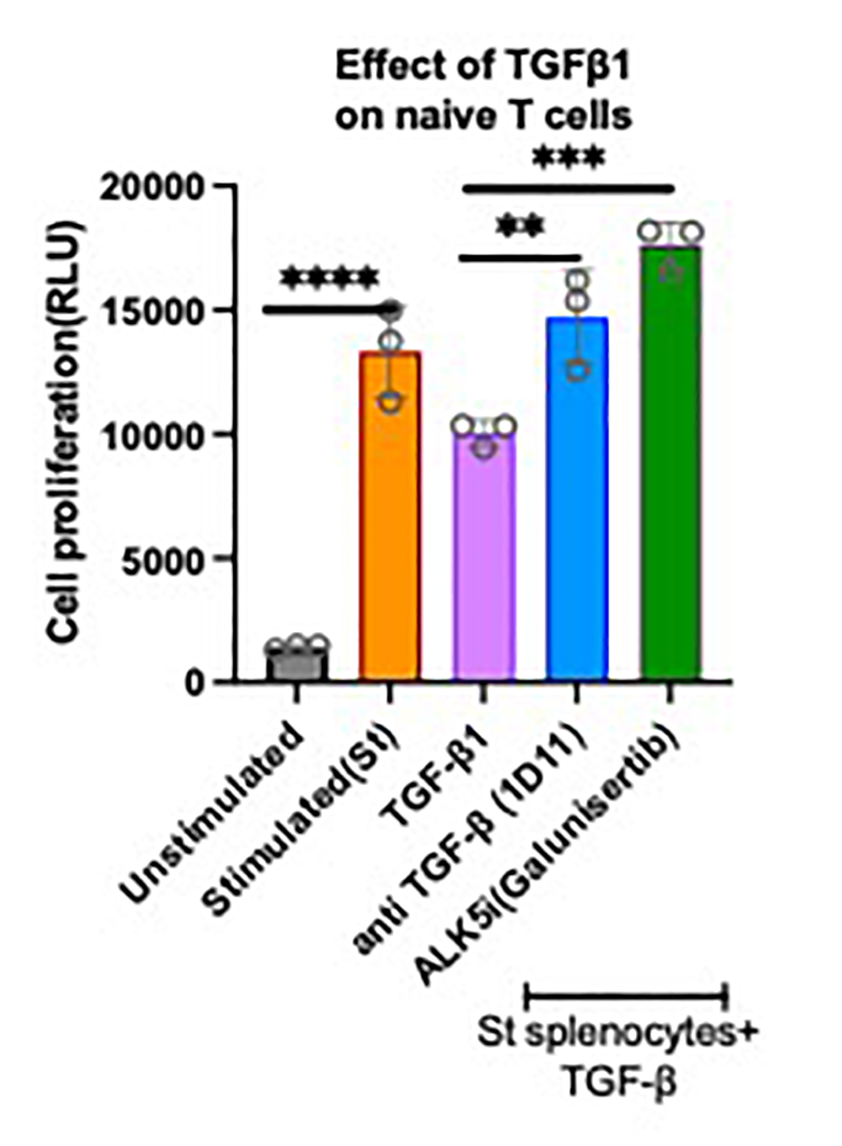

Supplement: Supplemental Material [file KONI_A_2330194_SM7783.zip › SupFig1C..tiff]

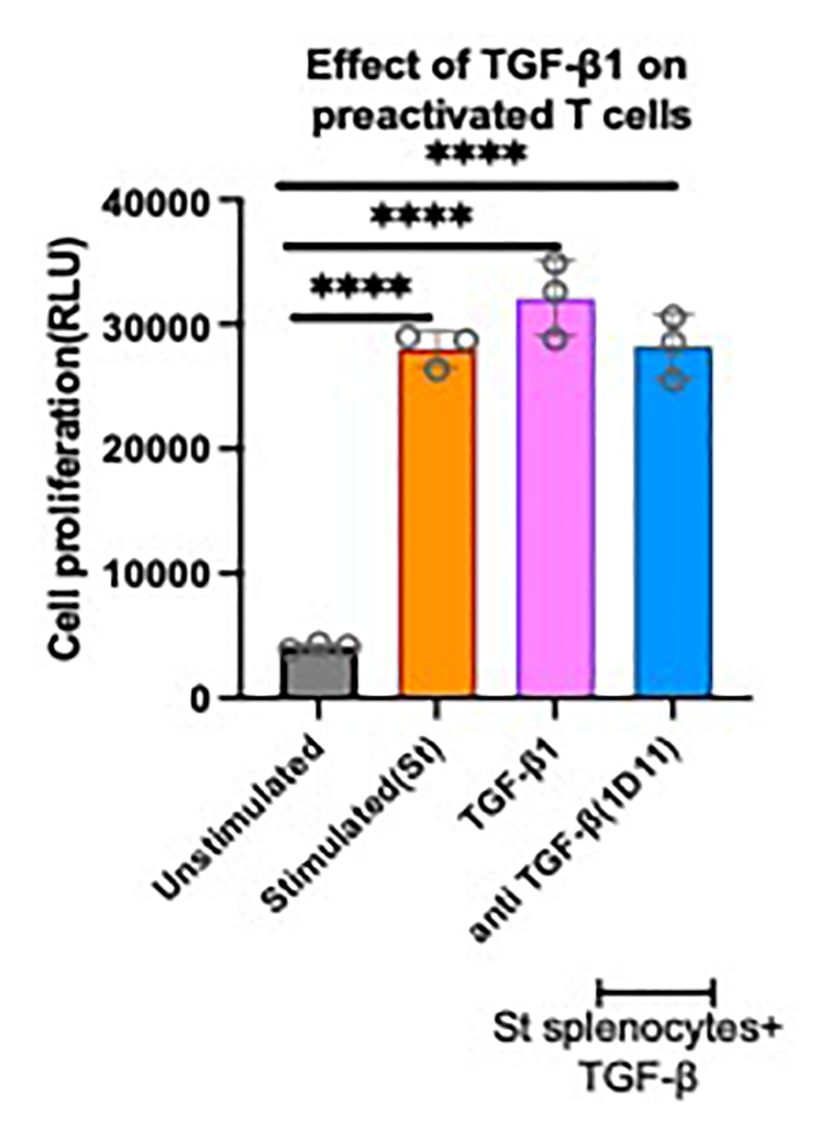

Supplement: Supplemental Material [file KONI_A_2330194_SM7783.zip › SupFig1D..tiff]

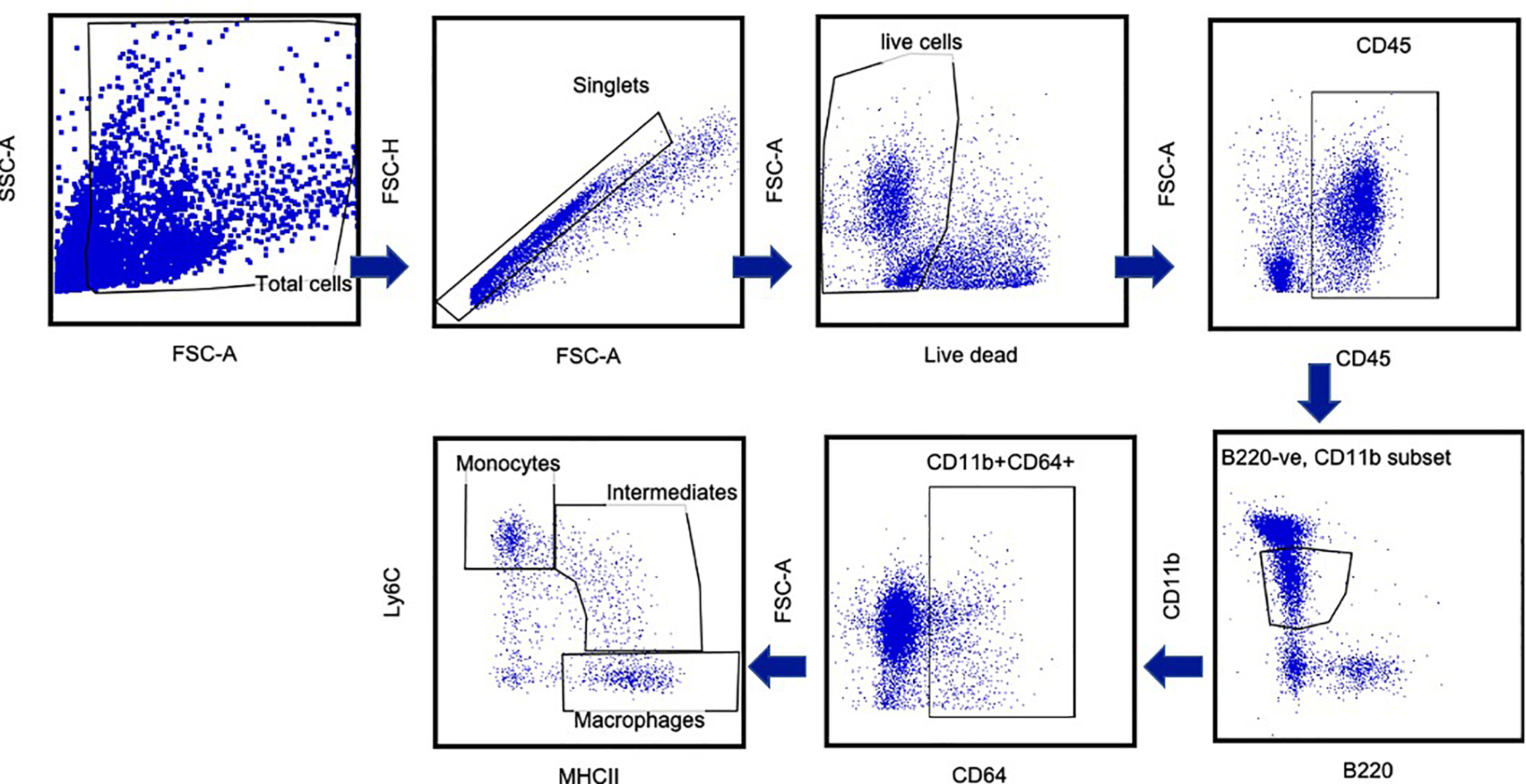

Supplement: Supplemental Material [file KONI_A_2330194_SM7783.zip › SupFig2A..tiff]

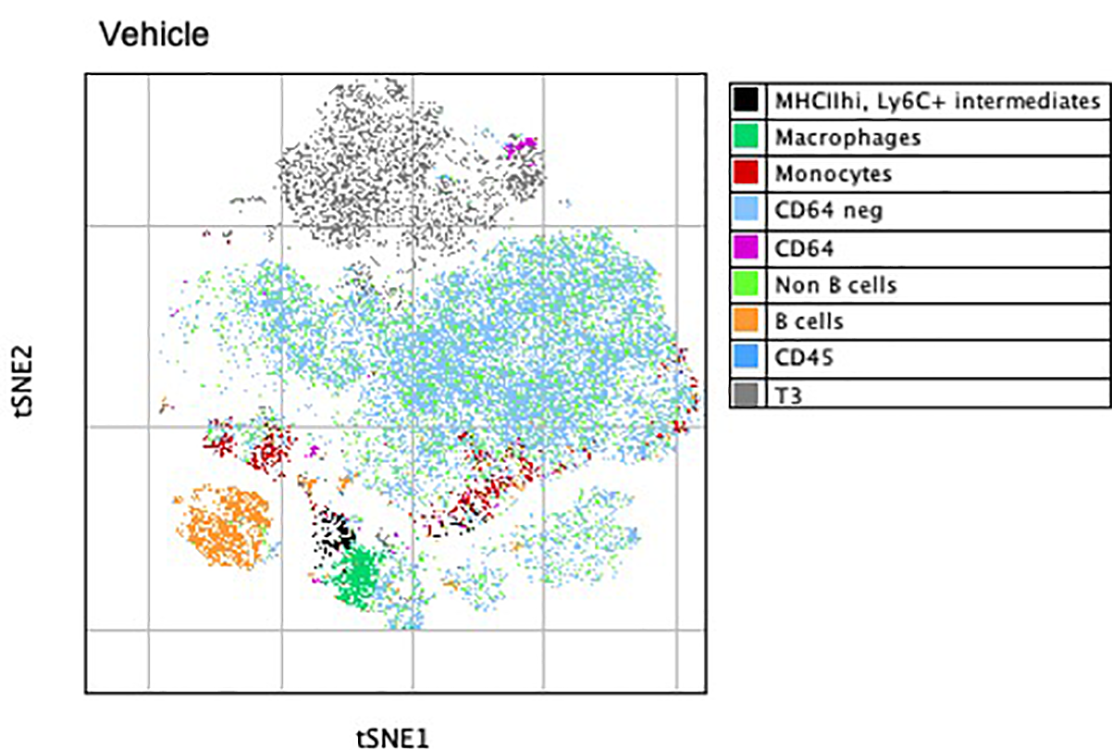

Supplement: Supplemental Material [file KONI_A_2330194_SM7783.zip › SupFig2B..tiff]

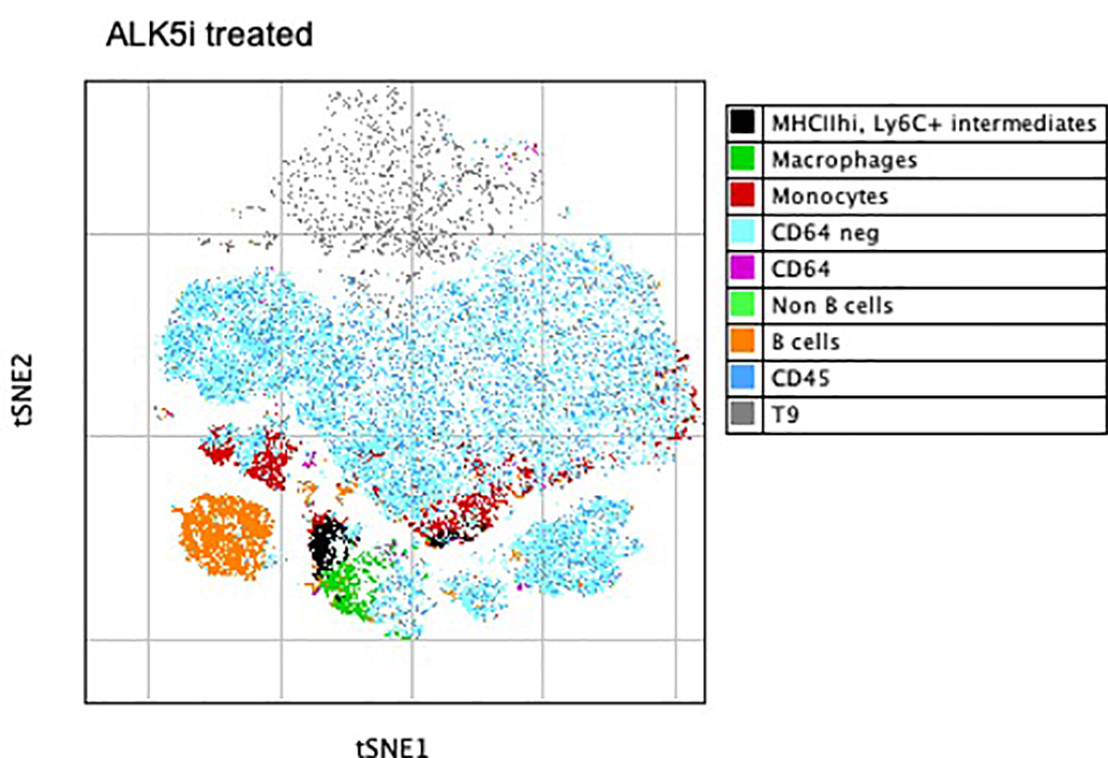

Supplement: Supplemental Material [file KONI_A_2330194_SM7783.zip › SupFig2C..tiff]

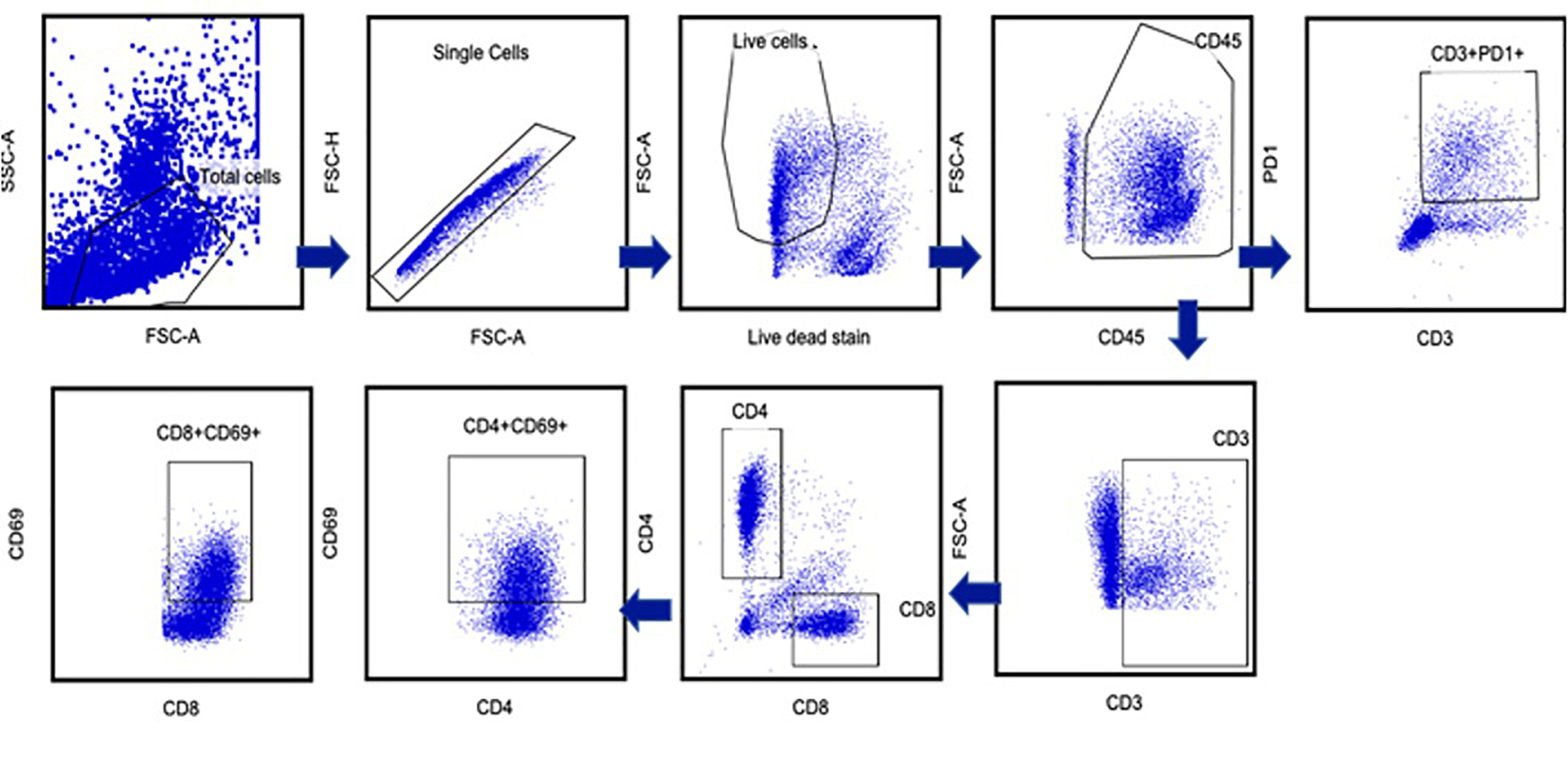

Supplement: Supplemental Material [file KONI_A_2330194_SM7783.zip › SupFig3..tiff]

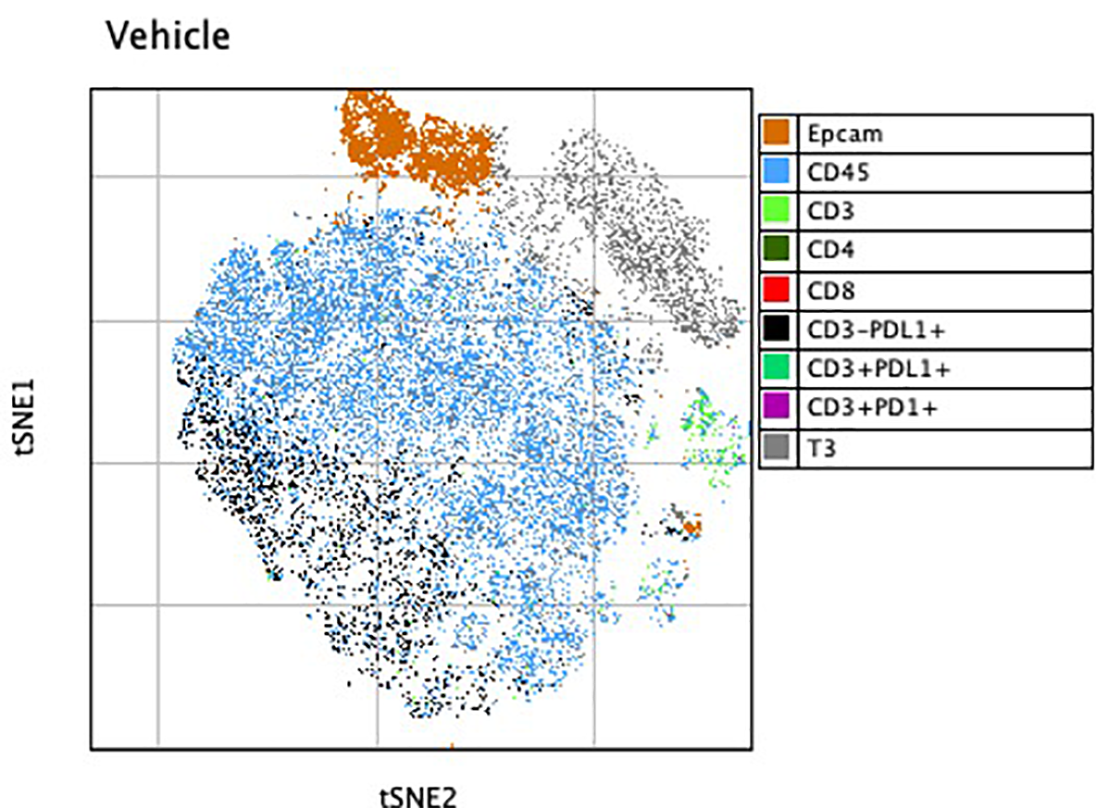

Supplement: Supplemental Material [file KONI_A_2330194_SM7783.zip › SupFig4A..tiff]

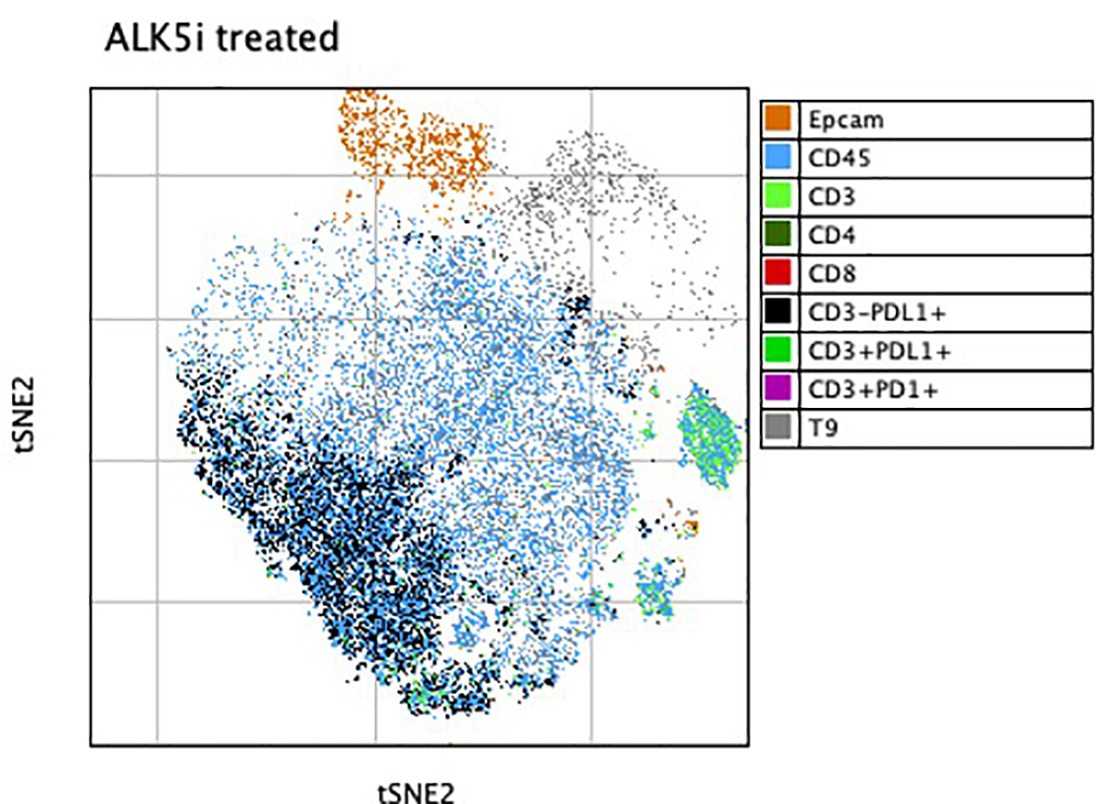

Supplement: Supplemental Material [file KONI_A_2330194_SM7783.zip › SupFig4B..tiff]

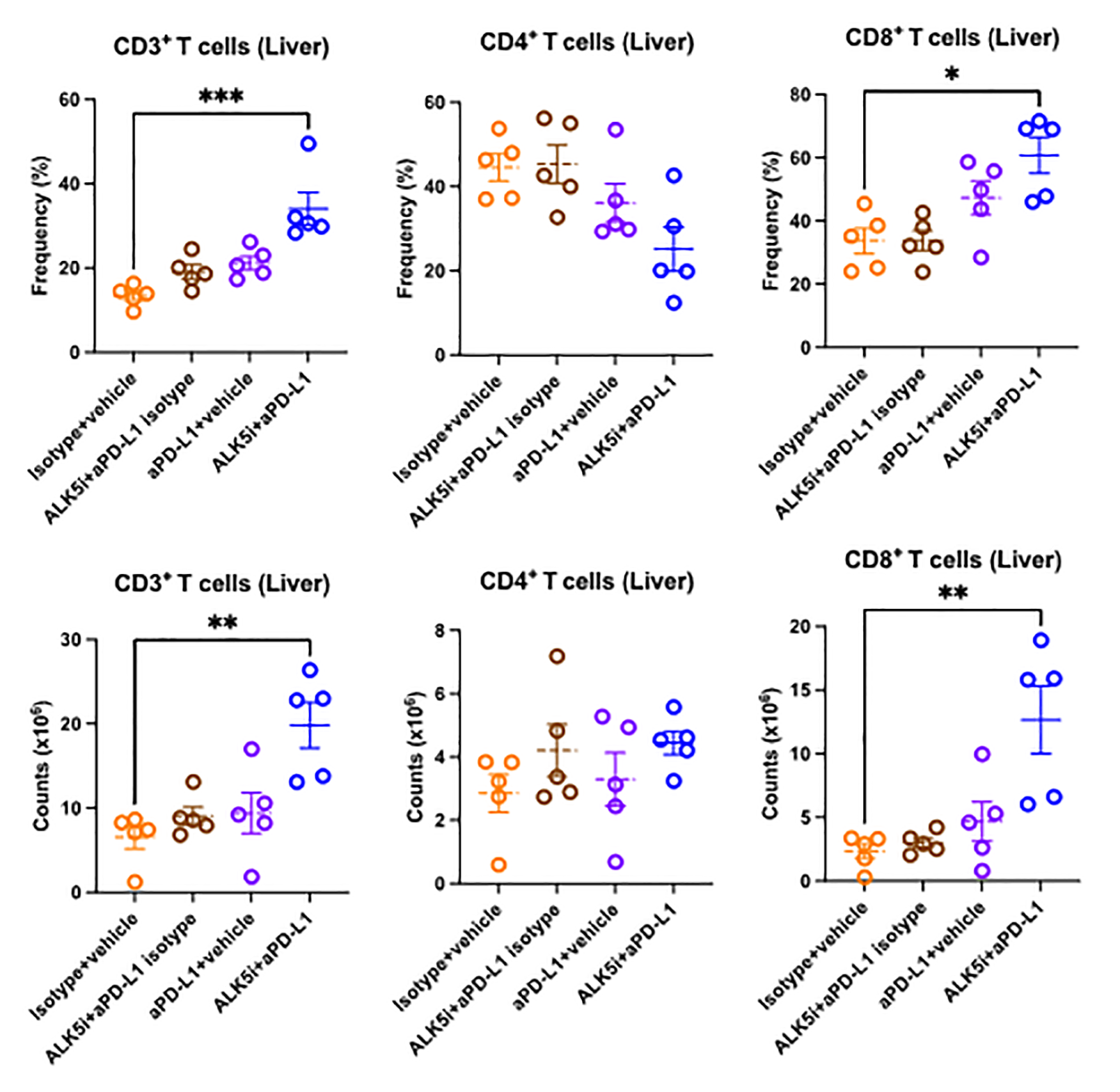

Supplement: Supplemental Material [file KONI_A_2330194_SM7783.zip › SupFig5A..tiff]

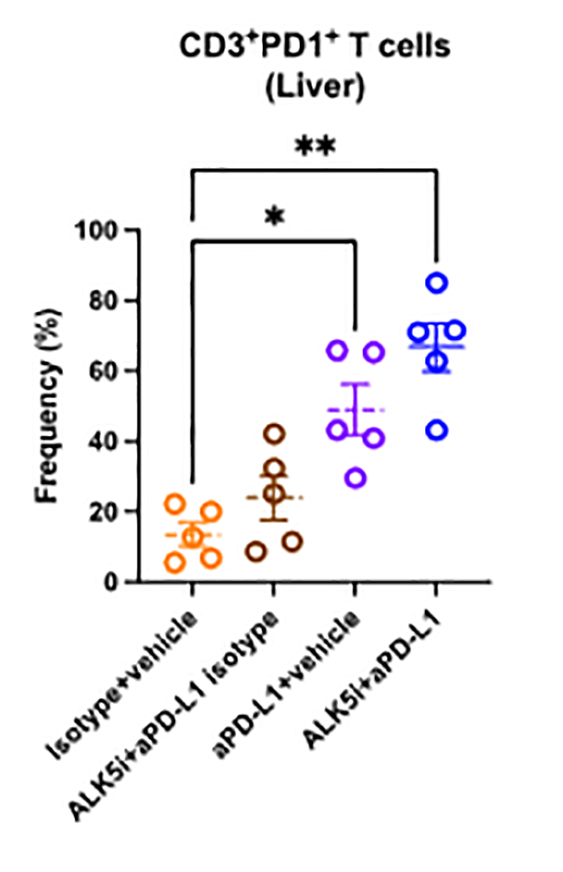

Supplement: Supplemental Material [file KONI_A_2330194_SM7783.zip › SupFig5B..tiff]

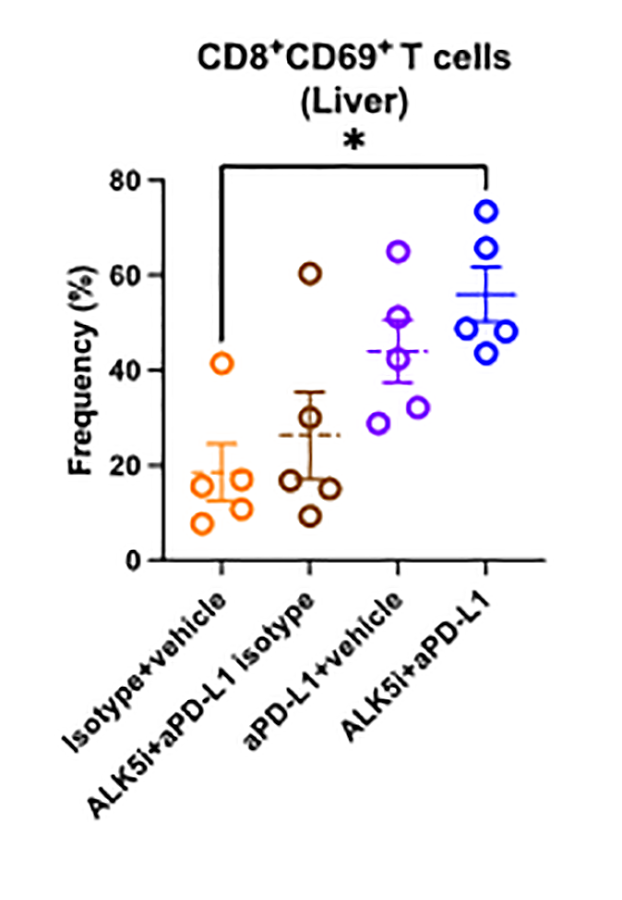

Supplement: Supplemental Material [file KONI_A_2330194_SM7783.zip › SupFig5C..tiff]

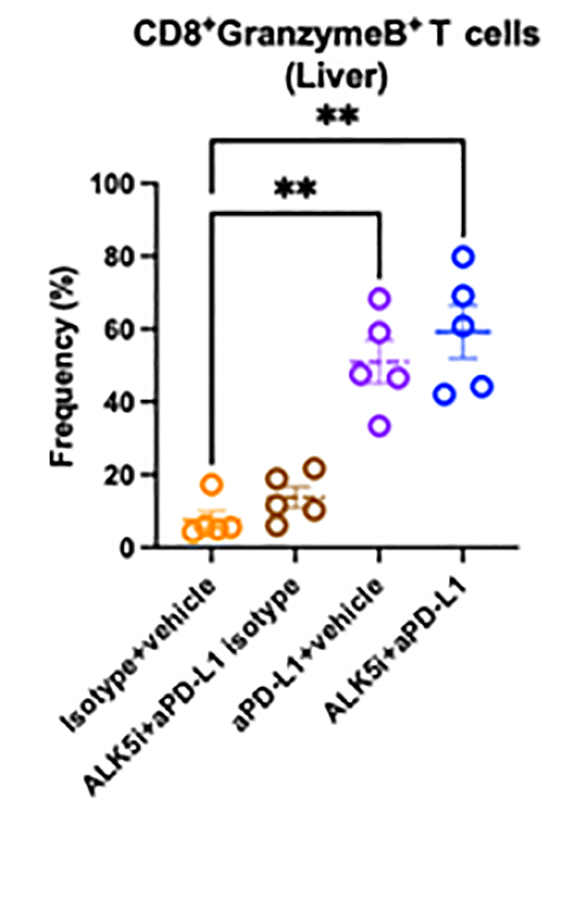

Supplement: Supplemental Material [file KONI_A_2330194_SM7783.zip › SupFig5D..tiff]

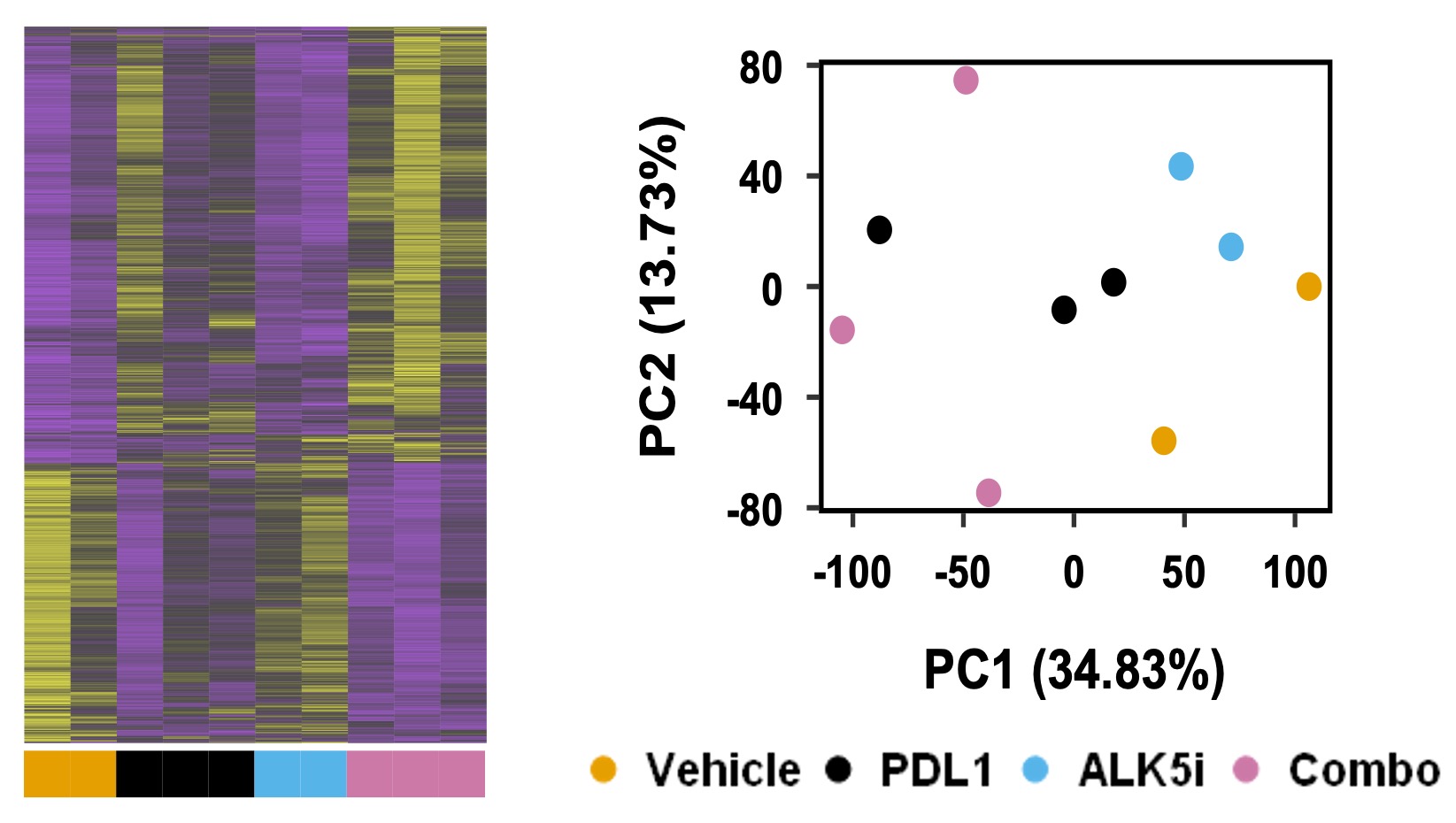

Supplement: Supplemental Material [file KONI_A_2330194_SM7783.zip › SupFig6A and 6B.jpg]

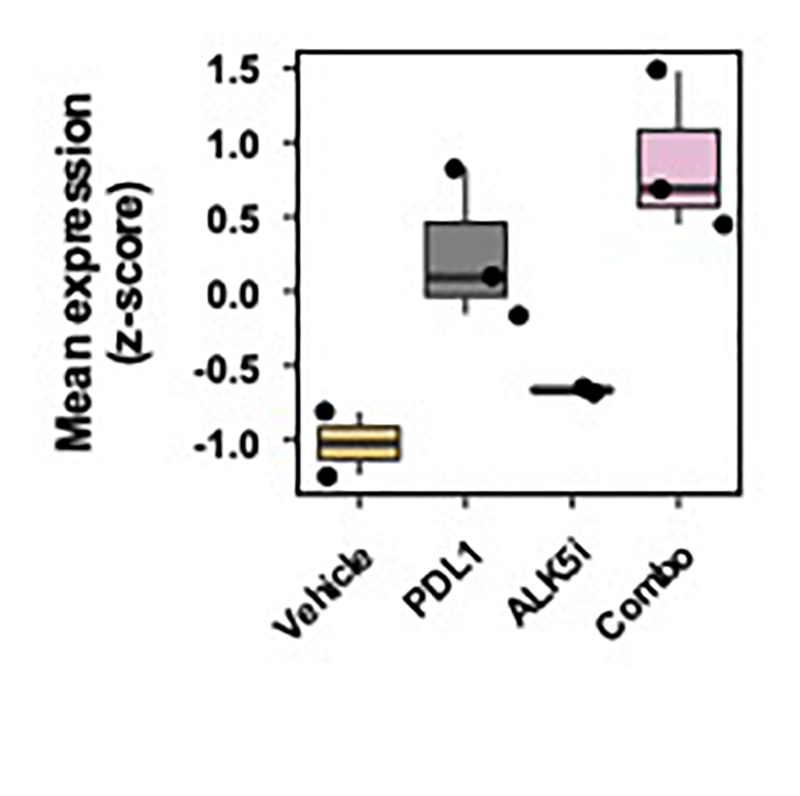

Supplement: Supplemental Material [file KONI_A_2330194_SM7783.zip › SupFig6C..tiff]

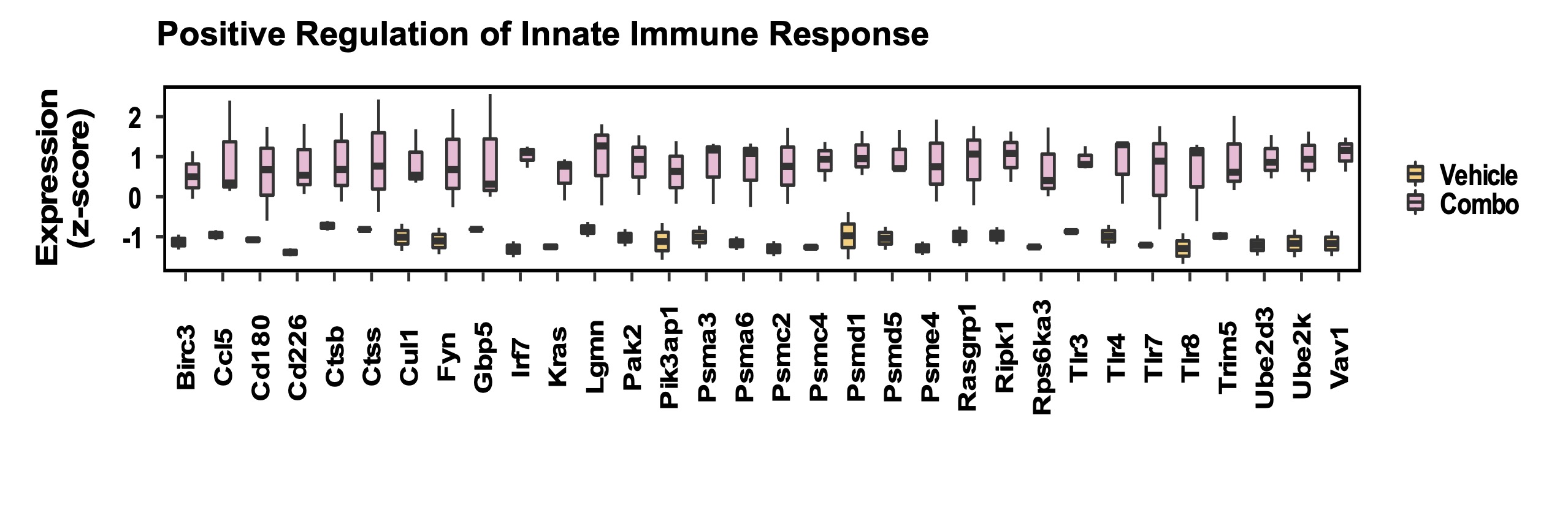

Supplement: Supplemental Material [file KONI_A_2330194_SM7783.zip › SupFig6D.jpg]

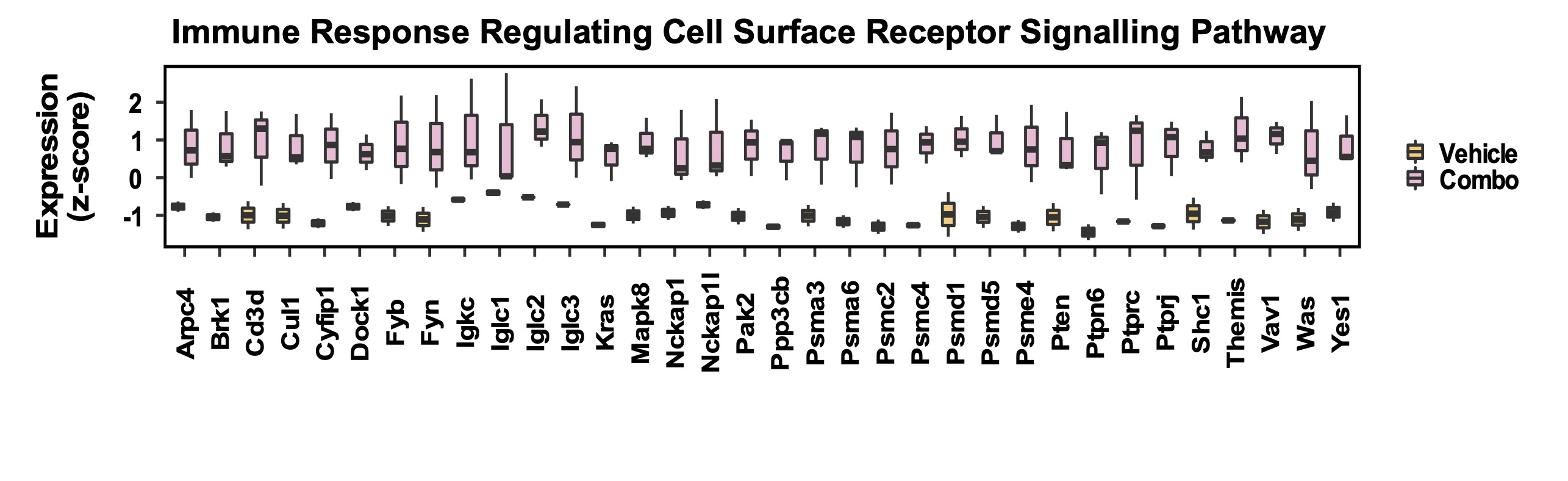

Supplement: Supplemental Material [file KONI_A_2330194_SM7783.zip › SupFig6E.jpg]

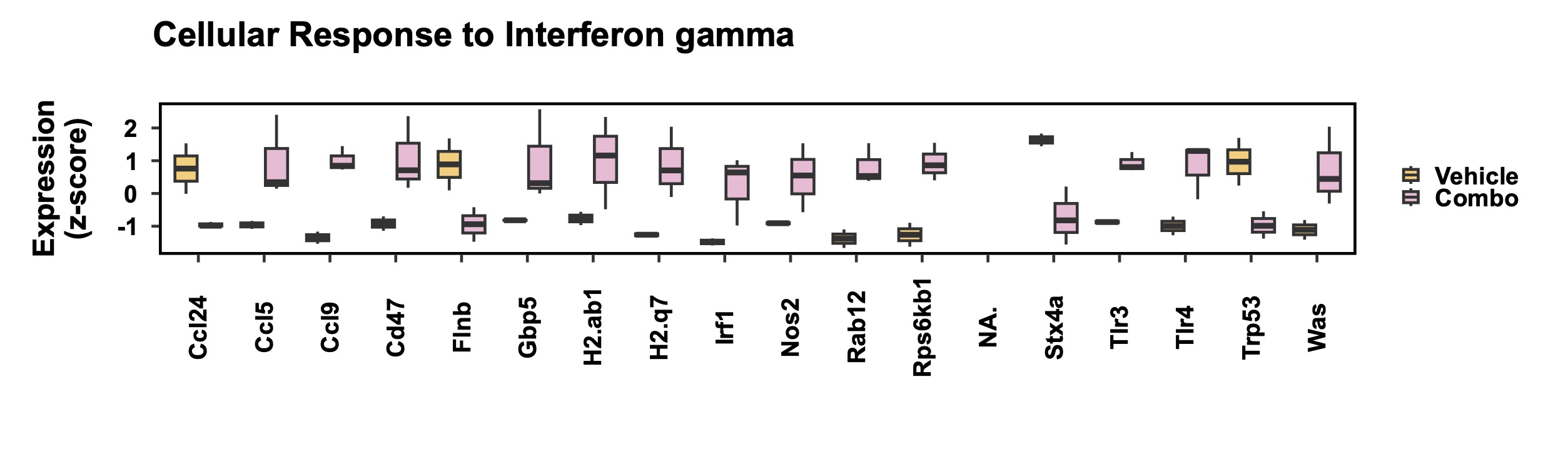

Supplement: Supplemental Material [file KONI_A_2330194_SM7783.zip › SupFig6F.jpg]

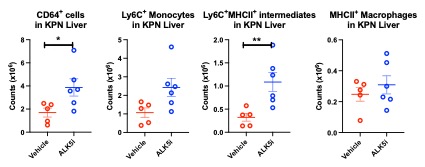

Supplement: Supplemental Material [file KONI_A_2330194_SM7783.zip › SupFig2D.jpg]
